# Experiences, perspectives and preferences for home-based palliative and supportive care: a qualitative study of individuals with heart failure and their carers

**DOI:** 10.1186/s12904-026-02097-x

**Published:** 2026-04-27

**Authors:** Madhurangi Perera, William Parsonage, Patsy Yates, Gursharan K. Singh

**Affiliations:** 1 Centre for Healthcare Transformation, Faculty of Health, Queensland University of Technology, 88 Musk Avenue, Kelvin Grove, Brisbane, QLD 4059 Australia; 2 Cancer and Palliative Care Outcomes Centre, School of Nursing, Queensland University of Technology, Brisbane, QLD Australia; 3 Australian Centre for Health Services Innovation, Queensland University of Technology, Brisbane, QLD Australia; 4 Department of Cardiology, Royal Brisbane & Women’s Hospital, Brisbane, QLD Australia

**Keywords:** Supportive care, Heart failure, Home-based care, Palliative care, Qualitative, Preferences

## Abstract

**Background:**

Palliative and supportive care provided in the home for individuals with heart failure and their carers can improve quality of life, reduce symptom burden and hospital admissions. However, enabling this care in accordance with what matters to individuals living with heart failure and their carers remains elusive. This research aimed to explore the experiences, perspectives and preferences of individuals with heart failure and their carers on home-based palliative and supportive care.

**Methods:**

A qualitative study using semi-structured interviews was conducted with individuals with heart failure and carers, recruited from two tertiary care hospitals in Queensland, Australia.

**Results:**

A total of eleven individuals with heart failure and ten carers participated. Their age ranged between 40 and 84 years. Most participants were female (*n* = 14, 66.7%). The themes derived from thematic analysis centred on: (a) diverse strategies for seeking emotional support, (b) acknowledging the importance of carers, (c) effective information and communication, (d) the value of home-visits, (e) telehealth enhances care (f) a circle of care: health professionals and social services supporting the individual and carer, and (g) planning for future care.

**Conclusion:**

Insights into the perspectives, experiences and preferences of individuals with heart failure and their carers are critical to delivering patient and carer centred palliative and supportive care in the home. Enhancing the circle of care through better communication, supporting carers and utilising telehealth, is required to deliver palliative and supportive care in the home in accordance with what matters to these individuals.

**Supplementary Information:**

The online version contains supplementary material available at 10.1186/s12904-026-02097-x.

## Background

The number of individuals living with heart failure (HF) is rising [1]. Despite advances in therapies, many individuals living with HF experience high symptom burden, multiple hospitalisations and a poor quality of life [2]. In addition, caregiving burden is high among carers of individuals with HF [2, 3], affecting the wellbeing of carers and contributing to carer depression [4].

Palliative and supportive care is defined as “an approach that improves the quality of life of patients and their families who are facing problems associated with life-threatening illnesses. It prevents and relieves suffering through early detection, correct assessment and treatment of pain and other problems, whether physical, psychosocial, or spiritual” (World Health Organisation [5]. Palliative and supportive care is recommended for individuals with heart failure and can be provided throughout the course of their disease and across care settings [6, 7]. The provision of palliative and supportive care in the home is critical, as individuals with HF spend most of their time at home [6]. Additionally, some evidence demonstrates a sustained reduction in HF symptom burden when this care is provided in the home [2], as well as cost savings through a reduction in hospital admissions [8, 9].

Previous literature has highlighted the components of home-based palliative and supportive care in HF [10], however there is currently limited evidence as to which of these components are most important to individuals with HF and their carers. Understanding which components of home-based palliative and supportive care are important can optimise the provision of this person-centred care in the home [11] and facilitate improvements in symptom burden, quality of life and hospitalisations. The aim of this study is to explore the experiences, perspectives and preferences of home-based palliative and supportive care of individuals with HF and their carers.

## Methods

### Design

A qualitative exploratory design was used to gain insight into the perspectives, experiences and preferences of individuals living with HF and carers in relation to what is important for enabling home-based palliative and supportive care in HF. One-on-one semi-structured qualitative interviews with individuals with HF and carers were conducted to obtain an in-depth insight.

### Participants and recruitment

The participants in this study were: (i) individuals aged ≥ 18 years who are diagnosed with HF and had a cognitive and functional capacity to engage in a qualitative interview and (ii) carers of individuals with HF with a cognitive and functional capacity to engage in a qualitative interview. Individuals with HF who are awaiting cardiac transplant and/or persons with HF and carers who cannot converse in English were excluded from the study. The participant’s eligibility was determined by the site investigators (who are HF health professionals at two tertiary hospitals).

Participants were recruited through purposive sampling. Twenty-three individuals with HF and twenty carers were approached face-to-face by the site investigators with details of the study. Amongst this group, 15 individuals with HF and 13 carers consented and semi-structured interviews were conducted with 11 individuals with HF and 10 carers. The individuals who did not participate were unable to schedule a suitable time for the interview (*n* = 2) or did not respond after consent was obtained (*n* = 5). As interviews were conducted separately and not as an individual-carer dyad, individuals with HF did not have to have a carer who was willing to participate in the study to be eligible for recruitment, and vice versa.

### Setting

Palliative care is provided by government, private and not-for-profit organisations in Australia [12], across a wide variety of settings, including but not limited to specialist inpatient and community-based palliative care services, acute public and private hospital settings, residential aged care settings and in some instances, in the home setting [12]. While palliative care services are recommended for individuals with HF [13] access to supportive and palliative care services in this population occurs late in the illness trajectory [14, 15]. Care provision for individuals living with HF occurs through shared care in the hospital setting either as inpatient or outpatient clinics and in the community. Participants in this study are individuals receiving HF care at two tertiary, metropolitan hospitals in South-East Queensland, Australia.

### Data collection

Data collection occurred between September 2024 - February 2025. The semi-structured interview guide used in this study is provided in Supplementary material 1 and was informed by existing literature and a scoping review exploring components of home-based palliative and supportive care in heart failure [10]. Prior to each interview, demographic information on gender, age group, place of residence, marital status was collected, and if a carer, relationship to the individual with HF was collected. All semi-structured, qualitative interviews were conducted by the first author (MP) either in-person (participant’s home or hospital), online, or via telephone, as per the participant’s preference. The interviewer, a female and a PhD candidate with previous experience as a medical practitioner and public health professional in Sri Lanka, had trained in qualitative research by attending two online education courses. During interviews, notes were taken, and reflexive summaries were written following each interview to capture initial impressions and potential influences on data collection. The interviewer’s professional and cultural background may have shaped interactions with participants and the interpretation of their responses. To enhance validity and credibility and to minimise potential bias, the interviewer had regular meetings with the research team to discuss insights and critically reflect on the data. No other individuals were present during the interviews. Among the 21 interviews that were conducted, one interview was repeated per the request of the participant, who was initially interviewed over the phone and selected to be interviewed again in-person. The first interview was discarded from analysis.

A semi-structured interview guide with open-ended questions was developed from the findings of a scoping review on components of home-based palliative and supportive care [10] and input from the research team. Following the first three interviews, the interview guide was refined iteratively for clarity and comprehensiveness. The questions which were refined for clarity include:

1. We know managing your/ your loved one’s symptoms at home by yourself/your loved one is a component of supportive care. What do you think about this?
2. Planning for your/ your loved one’s care is a part of supportive care. Looking back on the care you received, what do you think would have been more helpful to plan your care?
3. What is your understanding of what might happen in the future?

Each interview lasted between 45 and 90 min and interviews were digitally audio-recorded and transcribed verbatim by MP. Field notes and summary memos were added in square brackets within or after quotes. None of the participants were known to the interviewer. Data collection continued until coding saturation was reached [16, 17], which occurred after a total of 21 interviews. Several criteria was used to determine coding saturation in this study, defined as the point at which no new codes emerged from the data [18]. A pre-specified goal of coding saturation guided the process, where iterative data collection and analysis allowed the researcher to identify recurring codes across interviews [18]. Purposive sampling was used, with an initial target of 15 participants per group (individuals with HF and carers), based on literature recommending 10–20 participants per group for exploratory studies using thematic analysis [17]. Throughout data collection, the research team had regular discussions to review codes and assess saturation [18]. After nine interviews per group, no new codes were observed; two additional interviews per group were conducted to confirm saturation. Due to logistical constraints, only one additional carer interview was possible, and the research team reached consensus that the total of 21 interviews was sufficient to achieve code saturation [17].

### Data analysis

Inductive thematic analysis was used to explore perspectives, preferences and experiences towards home-based supportive care in the context of HF. The analysis was conducted from an essentialist-realist perspective, aiming to describe and interpret participants’ accounts as direct reflections. The analysis focused on semantic (surface-level) meanings and did not involve latent or theory-driven interpretation. An essentialist-realist perspective was chosen because the research aim, which was to explore the experiences, perspectives and preferences of individuals with HF and their carers regarding home-based palliative and supportive care, with the goal of closely capturing the participants’ voices as they experienced them. A semantic approach allowed us to identify participants experiences and preferences directly from the data without interpreting underlying assumptions or broader social constructs.

The six steps to data analysis, as described by Braun and Clarke [19] guided the analytic approach: (1) familiarisation of data which involved listening to the audio recordings, manually transcribing the interviews and re-reading the transcripts, (2) generating codes which involved line-by-line coding using an inductive approach and then reviewing alignment of the quotes and codes, (3) searching for themes, which involved sorting codes and identifying relationships between codes to search for themes, (4) reviewing the themes iteratively, (5) defining and naming the themes and.

6) producing the final report. Steps 1–6 was completed by the first author (MP), with input from the research team (GS, PY and WP) sought during steps 2–6.

### Trustworthiness and analytical rigour

Credibility, dependability and confirmability were established to ensure trustworthiness, in accordance with the COnsolidated criteria for REporting Qualitative research (COREQ) checklist, containing 32 items. Credibility was supported through member checking, where the participants were given the opportunity to read the transcribed interviews to verify accuracy, and through regular discussions with the research team [20]. Analytical rigour was ensured by maintaining a detailed audit trail of coding and analytic decisions and achieving consensus on themes with the researcher team. Reflexive summaries and notes taken during the interviews enhanced dependability and confirmability to ensure that the findings were grounded in the perspectives of the participants, as opposed to the researcher’s assumptions [20].

### Ethical considerations

The ethics and governance approvals were obtained from Metro North Research and Governance Office (HREC/2024/MNHB/99662) and administrative approval was obtained through Queensland University of Technology Human Research Ethics Committee. Prior to commencing data collection, written informed consent was obtained from all study participants. All names or places of work were removed from the data and participants were allocated pseudonyms (where individuals with HF are depicted as ‘P’ and carers are depicted as ‘C’) to maintain confidentiality and anonymity.

## Results

### Study participants

The demographic characteristics of the 21 study participants are shown in Table 1. Of the 21 participants, 11 (52%) were individuals with HF and 10 (48%) were carers. There was a total of 14 (70%) females, and all 10 carers were female. Among individuals with HF, 27% (*n* = 3)were aged 75–79 years, while 40% of carers (*n* = 4) were aged 55–59 years. Most study participants (*n* = 20, 95%) resided within the hospital’s service catchment.

**Table 1:** Demographic characteristics of study participants

Number of participants	Individuals with HF	Carers
	11 (52%)	10 (48%)
Age range (yrs)		
40–44	-	2 (20%)
50–54	2 (18%)	-
55–59	2 (18%)	4 (40%)
60–64	1 (9%)	2 (20%)
65–69	2 (18%)	-
70–74	-	1 (10%)
75–79	3 (27%)	1 (10%)
> 80	1 (9%)	-
Sex		
Female	4 (36%)	10
Male	7 (64%)	-
Marital status		
Single	2 (18%)	2 (20%)
Married	7 (64%)	6 (60%)
In a de facto relationship	-	1 (10%)
Divorced	1 (9%)	1 (10%)
Widowed	1 (9%)	-
Relationship to the person with HF (carers only)		
Spouse	-	4 (40%)
Child	-	4 (40%)
Other - Parent	-	2 (20%)

*HF *Heart failure

### Themes

The analysis from the qualitative interviews with participants generated 7 themes.

1. Diverse strategies for seeking emotional support
2. Acknowledging the importance of carers
3. Effective information and communication
4. The value of home-visits
5. Telehealth enhances care
6. A circle of care: Health professionals and social services supporting the individual with heart failure and carer
7. Planning for future care

The final themes, subthemes, and illustrative quotes are presented in Table 2.

**Table 2:** Summary table of themes, subthemes and illustrative quotes

Theme	Subtheme	Illustrative quote
Theme 1 - Diverse strategies for seeking emotional support	Emotional responses	“Over the years and I’ve learned to cope…just sort of grin and bear it” (P 4, age: 75–79)
	Managing emotional responses	“If they’re [patients] having a serious chronic condition, you don’t know, the possibility of dying on this and this, you need some sort of counselling” (P 12, age:55–59).
Theme 2 - Acknowledging the importance of carers	The many roles of carers	“I was just trying to coordinate doctor’s visits and all the other things that come with life, you know, hairdresser appointments, all of that sort of stuff. Because she wasn’t driving by this stage… Yes, doing all of that sort of stuff for her”. (C 3, age:60–64)
	Overcoming the challenges of caregiving	“I don’t think anything could make a difference because I do things the way I know my mom needs them. And it’s not easy. It’s easier since I rejigged my working life, but I pay for that financially, only working four days a week. I just think that’s just the way it is”. (C 9, age: 55–59)
	The importance of communicating with and informing carers	“[Nurse practitioner and health professional at HF clinic in Hospital A] went through the medication as to you know, why this one’s so important and why that one’s so important and how they work together and things like that. So, it gave me a better understanding as to this medication that I’m giving him and what it’s actually doing for him.” (C 7, age: 55–59)
Theme 3 - Effective information and communication	Experience of information delivery on HF	“At the beginning, it was a minefield…So I felt that sometimes I was getting bombarded with stuff. Bam, bam, bam! And then…other things are going on at the same time… I was coming in here [hospital A] three times a week for different things, not just for this.” (P 13, age: 65–69)
	Disjointed communication between health professionals	“We’re doing everything we’re asked to do. We go to all the appointments, he does all the tests, and it’s like nobody’s speaking to each other. Um, so that part can get frustrating” (C 7, age: 55–59)
	Preferences for the mode of information delivery	“I’m an old-fashioned person. I like the paper. I like hard copy…and [spouse] he’s old fashioned too, so if he wants to learn about something he’d much rather read it”. (C 8, age: 60–64)
	Good communication involved transparency and the opportunity for clarification	“… I have faith in mom’s new GP, so just somebody to be able to ask a definitive question to and get an answer whether it be, should this be happening or yeah that sort of thing” (C 9, age:55–59)
Theme 4 - The value of home-visits	Supporting daily life activities	“The first one [Occupational therapist] came when I was having the dizzy spells, and they got me the wheelie walker…They [Occupational therapists] found that sliding door was too high for me … I couldn’t get from the front veranda down into the carport to get in the car. She [Occupational therapist] measured up and made me a step to the carport and the ramp for the back of the house”. (P 4, age: 75–79)
	Medical management in the home	“A lot of them are just medical issues … unless a doctor visited him at home, I don’t think there’s much else that could be done because it was like, you know, a urinary urgency and urinary tract infections and stuff… I can’t think of anything else apart from medical support that he would need or a nurse. I mean … that’s fantasy land stuff; I know that can’t happen but yeah.” (C 2, age: 40–44)
Theme 5 - Telehealth enhances care	Telehealth provides support and reassurance	“It builds confidence, but it decreases anxiety and those two combined are just a magic combination” (C 9, age 55–59).
	Nurse practitioners are well placed to provide telehealth	“I think it was also [Nurse Practitioner] that also said, if there was ever anything that I needed or would like to know more about or inquire about more information in order to help [Name of patient] from them, for me to get in touch any time” (C 5, age 40–44)
Theme 6 - A circle of care: Health professionals and social services supporting the individual with heart failure and carer	Continuity of care delivery	“Although once again, as I said earlier, the fact that they’re [Heart support service] there and they’re we’ve been seeing them regularly, is enormous. I mean, it’s just such a relief. Just a huge relief” (C 8, age:60–64).
	GP’s and nurse practitioners as care coordinators in a multidisciplinary team	“…Doctor D [new GP] has contacted just about everybody that mom has come into contact with relating to heart failure at Hospital X and other issues at regional hospital Y. And she [new GP] has nailed it {stressing each word}! This woman is wonderful! (C 9, age: 55–59)
	Community supports	“When he could play sport and golf, he had outside interests where there’s not been a lot of input into that component of this rehab, like what he can do, what activities are available in the community, what community groups he could potentially link into. He hasn’t had any of that input, which I think would be really important as part of his rehab. And still allowing him to see that he can still do things he can still interact with people and, you know, still have that component of interactions, not just me that he needs to have near him for support” (C 12, age: 55–59)
Theme 7 – Planning for future care	Various levels of planning had occurred	“…I mean, I don’t think we’re anywhere near there [severely ill and symptomatic] yet. So, there’s been no discussion of any plan, just regular checks with the GP and we’ll come and see the specialist and if there’s any changes, they’ll deal with them as they occur. That’s sort of it thus far” (C 8, age: 60–64)
	Quality of life matters	“Quality of care over quantity of care means an awful lot… And the quality of life is not going to be worth a cracker, then there’s no point in mum existing. Because that’s not what she wants” (C 3, age: 60–64).

#### Theme 1 - Diverse strategies for seeking emotional support

##### Emotional responses

Individuals living with HF and carers experienced various emotions associated with their HF illness. These emotions included “…*shock! Denial!”* (P 4, age: 75–79), and for some, a sense of acceptance or trying to persevere stoically.

*“Over the years and I’ve learned to cope…just sort of grin and bear it”* (P 4, age: 75–79).

Participants also highlighted the unpredictability of HF, which led to feelings of fear and anxiety when going about their day-to-day activities.

*“… because it is kind of scary. Well*,* actually*,* it’s a lot scary*,* when you’re asymptomatic*,* [because] you just don’t know what’s going to do to set it off. You live in fear. You want to exercise. You’re just afraid of killing yourself to do so”* (P 15, age:60–64).

##### Managing emotional responses

The ways in which individuals with HF and carers preferred to cope with their emotions varied. While some participants preferred counselling and seeking professional psychological support, others preferred to share their thoughts with family and close friends or utilise mind-body practices. Counselling was preferred to navigate the uncertainty of the HF illness trajectory.

*“If they’re [patients] having a serious chronic condition*,* you don’t know*,* the possibility of dying on this and this*,* you need some sort of counselling”* (P 12, age:55–59).

This preference was also shared by carers who were receptive to receiving counselling for feelings of worry and uncertainty. Carers acknowledged that counselling could “*soothe and help our sense of uncertainty at our fingertips*.” (C 2, age:40–44). Extending the psychological support for the whole family was also preferred as participants noted the involvement of family in the provision of care for the patient.

*“So that’s [psychological support] something that I think would be very beneficial. That way*,* the whole family and the support in the home are being looked after in order to support him [individual with HF]. And if something happened to another family member*,* like*,* say*,* if there was with me*,* we’ve got all the tools to be able to [self-manage] I think it’s it’ll [psychological support for family] be beneficial”.* (C 5, age: 40–44)

Some participants preferred that their emotional needs were addressed by their family or close friends, rather than seeking professional help for counselling.

*“‘that’s what I’ve got you [spouse]*,* my mates for’. He’s [individual with HF] only got about two very close mates that he’ll talk to about anything like that*,* and even then I don’t think he tells them everything*,* but*,* um*,* he seems good in that way”.* (C 7, age: 55–59)

Some individuals noted counselling wasn’t something they preferred due to their personality traits such as a lack of willingness to share their psychological concerns with others.

*“No*,* no*,* he’s not like that at all [when asked if psychological support would benefit her spouse]. No*,* it just very*,* very inward! Very! It all goes in*,* it stays there yeah… he’s [spouse the patient] always been very guarded and it’s gonna stay that way.”* (C 8, age: 60–64).

Some participants relied on their spiritual and religious beliefs to navigate their emotional experiences.

*“I always expect unexpected*,* yeah*,* ’cause I do a lot of talking to God every day…I’m thankful to be alive”* (P 6, age: 50–54).

Similarly, participants utilised other spiritual tools such as mind-body practices to address their feelings and emotions.

*“So that’s when my sort of mental*,* mainly emotional health kicks in and I’m just gonna take a step back and breathe”* (C 5, age: 40–44).

#### Theme 2 - Acknowledging the importance of carers

##### The many roles of carers

Carers played several key roles in the life of the individual with HF, enabling them to navigate their illness. The role of carers was considered as vital to the wellbeing of individuals with HF. In some cases, carers were the first point of contact for individuals with HF during emergency situations, as opposed to health professionals.

*“I’d say he [person with HF - father of the carer] probably would call one of us [if person with HF were to feel unwell]. (C 6*,* age:55–59)*

Carers were pivotal in supporting individuals with HF, by monitoring symptoms at home and providing medical management.

*“… I’ll check his ankles and if they’re a bit swollen*,* then we start on his fluid retention medication for a few days.” (C 2*,* age: 40–44)*.

Carers also acted as an information liaison between the health professional and the individual with HF. This was due to carers’ ability to retain information relating to signs and symptoms and then communicate this to health professionals to enable a change in medication management.

*“So that’s why I found that I needed to be with my husband on all the appointments because I knew that he’d be like*,* ‘yeah*,* everything’s all good’*,* whereas I can say ’he’s had a bit of a dizzy spell’ or and that’s how we worked out too last week*,* um*,* because his high blood pressure was just way too low. So one of the medications was too strong for his heart*,* so we’ve come down a little bit”.* (C 5, age: 40–44)

In addition to these roles, carers also played a key role in activities of daily living due to the functional limitations of the individual with HF.

*“I was just trying to coordinate doctor’s visits and all the other things that come with life*,* you know*,* hairdresser appointments*,* all of that sort of stuff. Because she wasn’t driving by this stage… Yes*,* doing all of that sort of stuff for her”.* (C 3, age:60–64)

Some carers also spoke about the difficulty of having the lead role in decision-making when the individual with HF hands over their agency to the carer.

*“I talked to him about it and he goes*,* “Mate*,* you’re talking mumbo jumbo to me. I don’t know what you’re talking about. I’m just trusting that you you’re giving me the right stuff and that you’ve spoken to the right people”* (C 7, age:55–59).

##### Commitment to caregiving despite its challenges

Carers highlighted that the many roles they played in supporting the individual with HF led to challenges around feeling “*overwhelmed*” (C 2, age (40–44) and navigating components of life outside of caregiving, including work and financial constraints.

*“I don’t think anything could make a difference because I do things the way I know my mom needs them. And it’s not easy. It’s easier since I rejigged my working life*,* but I pay for that financially*,* only working four days a week. I just think that’s just the way it is”.* (C 9, age: 55–59)

Familial support was vital to both individuals with HF and carers, as they offered help with day-to-day activities, as well as management of HF.

*“We’re lucky*,* we’ve got a son and a daughter. Our daughter is 10 minutes down the road and she’s been very good…And her husband is fantastic. He’s even been to the hospital to pick [Name of spouse] up”* (P 4, age: 75–79).

*“What comes to mind is probably you know*,* like my whole family yeah…because that’s what she [spouse] said*,* ‘look*,* you guys [Children] have to learn how to do this medication too*,* just in case’ … so that’s the support*,* I suppose*,* my kids…”* (P 6, age: 50–54).

Despite the acknowledgement of the challenges, many carers perceived and accepted their role of caregiving as something that can only be done by them and took pride in caring for their loved one.

*“For the next 100 years and I would do it even more*,* if I had to”* (C 2, age (40–44).

Several participants described a reciprocal caregiving relationship with their spouse and that working together as a team was supportive of enabling an overall wellbeing and improved medication adherence.

*“Yes*,* I love it! We work really well as a team that’s what I love so much with him is that I’ll get frustrated because he’ll wake me up to give my medication and I’ll do the same. I’m like*,* ‘Oh*,* what’s the point of taking this? And there’s no changes {laughs}. And then he’s the one that picks me up*,* so it’s- It balances really well”* (C 5, age:40–44).

##### The importance of communicating with and informing carers

Carers emphasised that it was important that they are communicated with, and provided with information, given they had a key role in providing care to the individual with HF.

*“[Nurse practitioner and health professional at HF clinic in Hospital A] went through the medication as to you know*,* why this one’s so important and why that one’s so important and how they work together and things like that. So*,* it gave me a better understanding as to this medication that I’m giving him and what it’s actually doing for him.”* (C 7, age: 55–59).

Some carers preferred to be given the given the opportunity to communicate with health professionals on behalf of the individual with heart failure to avoid challenges in receiving the medical support needed.

*“…I feel like I’m not getting listened to because then I’ll start explaining ‘look*,* since the last time we’ve been here*,* this has happened da da da da’*,* and then*,* you know*,* sometimes they’ll [emergency department health professional] listen to me*,* or sometimes they won’t. And then they’ll just ignore me and just try and ask [individual with HF] and he’s going ‘well*,* she just told you*,*’”* (C 7, age: 55–59).

Ensuring effective communication with carers could be facilitated through telehealth appointments, where some carers stated they were more relaxed.

*“when you talk about telehealth like if you can organise the telehealth meeting or appointment at the time the carer and the patient are there at together in the house where you know*,* something may trigger a question*,* where when you come to the hospital and you have all the stress and you know you’ve gotta get to the appointment*,* you might forget”* (C 12, age: 55–59).

However, whilst some carers wished to be more informed, they noted that this required permission and support for this to occur from the individual living with HF.

*“…as a carer I mean*,* I don’t get privy to that information [medical information that is available for all departments to see i.e. My Health record] unless it’s shared by my dad. Because he is an adult. He’s still in his right mind… I suppose if Dad wanted me to come to his doctor’s visits*,* that would be something I could do*,* and I could be more involved that way. But he still likes to think*,* he is still quite independent*,* and he can keep up with the doctor”* (C 6, age: 55–59).

#### Theme 3 - Effective information and communication

##### Experience of information delivery on HF

Participants’ experience of receiving information varied. Some individuals with HF noted that receiving information at the point of diagnosis was *a bit of a whirlwind”* (P 2, age: 50–54) and challenging, which in some instances was further complicated by other health concerns.

*“At the beginning*,* it was a minefield…So I felt that sometimes I was getting bombarded with stuff. Bam*,* bam*,* bam! And then…other things are going on at the same time… I was coming in here [hospital A] three times a week for different things*,* not just for this.”* (P 13, age: 65–69).

For other participants, their previous experience of receiving information on HF was comprehensive and not as overwhelming.

*“…they [health professionals at hospital] gave the booklet [on HF] out*,* I got one too when we came to the seminar thing… that they hand out*,* they’ve been fine. It’s not too overwhelming…So it’s [providing information] been done in an OK way*,* not frightening or anything”.* (C 8, age:55–59)

##### Disjointed communication between health professionals

A number of participants experienced communication challenges, where health professionals did not receive or review information in a timely manner, which led to fragmented care.

*“Communication downfall. Sometimes the GP don’t necessarily get the information in a timely fashion sometimes” (C 12*,* age: 55–59).*

*“[X Hospital] have always sent the correspondents through and usually via email. And there were things that were missed by her first GP. Just been missed”* (C 9, age:55–59).

This led participants to express frustration, despite diligently following their treatment plan:

*“We’re doing everything we’re asked to do. We go to all the appointments*,* he does all the tests*,* and it’s like nobody’s speaking to each other. Um*,* so that part can get frustrating”* (C 7, age: 55–59).

Involvement of multiple health professionals involved in care provision also sometimes led to conflicting information being provided, which led to confusion and a lack of confidence in health professionals.

*“And it’s confusing because … you’re going and seeing different people and you’re getting these different answers all the time you just lose your confidence*,* you don’t feel confident. I often wonder*,* there are like*,* talking about [Nurse practitioner C] clinic*,* right*,* you got [Nurse practitioner C] and the physios and the cardiologist*,* do they consult one another?… I don’t know but I just wonder if there’s communication between them”* (P 10, age: 75–79).

##### Preferences for the mode of information delivery

Many participants spoke about their preferences for how information about HF should be delivered, and this varied widely. Participants noted that they would prefer if information sessions for individuals with HF and their carers were made a requirement, so that they had a complete knowledge and understanding.

*“And so yeah*,* would it have been cool for someone be like ‘hey we see that you’re probably gonna be taking care of your dad*,*’ or then let’s just say dad didn’t have me and he was on his own doing this stuff*,* ‘hey*,* there’s a lot to know*,* we say that it’s mandatory. You need to come to this 5-hour seminar*,* and we need to give you the run through of everything so that you fully understand’. (*C 2, age: 40–44)

The preferred mode of information delivery varied from paper-based format such as brochures, online, or through face-to-face delivery. Some participants preferred a paper-based mode of information delivery as opposed to online information.

*“I’m an old-fashioned person. I like the paper. I like hard copy…and [spouse] he’s old fashioned too*,* so if he wants to learn about something he’d much rather read it”.* (C 8, age: 60–64)

Some preferences for paper-based format stemmed from participants being wary of inaccurate and misdirected information online and the need to be knowledgeable in using online technology to access information on HF.

*“… I’m quite conversant with online and I think you got to be so careful online because there is so much misinformation within the medical fields. Not a rabbit hole*,* an elephant hole”* (C 10, age: 70–74).

*“Only if they are tech savvy*,* with that equipment”* (C 3, age: 60–64).

Information delivered via in person sessions was also preferred by some participants as in-person interactions allowed participants to “*have a conversation with them [healthcare professionals] and ask them questions if need be…So good! You know*,* the seminars are*,* uh what do you call it*,* just more contact”.* (P 14, age: 65–69). Preferences for face-to-face information sessions were perceived as allowing more connection as opposed to other modes.

*“I’m a bit old school; I like to face to face. I like to actually sit down and talk to someone than do phone call… But that’s just me and [person with HF] is the same way because he just feels disconnected when it’s over the phone… whereas there’s more connection when you’re face to face…”* (C 7, age: 55–59).

Face-to-face delivery was also viewed as beneficial to enabling a comprehensive understanding of medication and management and the HF illness in a relaxed setting.

*“It takes a bit to process what’s going on and then someone sit down there*,* explain the situation…. Gently*,* maybe just have a cup of tea with somebody or something. Make it a friendly environment*,* not a stressful environment.”* (P 12, age: 55–59).

Regardless of the preferred mode of information delivery, having the ability to revisit the information was vital to enabling an understanding based on the individual’s preferred pace of processing information.

*“You know like Zoom or something like that so that I can ask questions and have maybe videos so that you know that I can go back and refer to it you know. As you know*,* sometimes it’s you*,* you listen*,* you watch something and then you need to go back and watch it again”* (P 8, age:55–59).

*“Personally*,* myself I prefer a brochure*,* I’m a very tactile person. I can sit there and I can read it*,* look at it and then go back over it and sort of make notes if I need to*,* just sort of query things”* (C 10, age: 70–74).

##### Good communication involved transparency and the opportunity for clarification

Participants spoke about their preference for transparent communication. They valued health professionals taking the time to explain and answer questions the individuals with HF and their carers may have in an honest and respectful manner.

“*So*,* they’ll [cardiologist] sit down or take the time to talk to you… and they wouldn’t hold back…they [cardiologists] were very honest. I appreciate honesty and not being talked down to.”* (C 10, age: 70–74).

The ability to seek clarification and definitive answers from health professionals was preferred as it fostered trust in health professionals

*“… I have faith in mom’s new GP*,* so just somebody to be able to ask a definitive question to and get an answer whether it be*,* should this be happening or yeah that sort of thing”* (C 9, age:55–59).

However, some participants spoke about wanting more time to communicate, to ask questions and seek clarification, with their healthcare professionals.

*“A bit more time with them [specialist] and being myself instead of having to zip it. They’ll give you 10 minutes to listen.”* (P 14, age: 65–69).

Additionally, participants noted that transparent communication with their healthcare professional involved communication in plain language, without the use of jargon

*“If you’re a non-medical person*,* it can be… quite [difficult] if the doctor isn’t able to explain it in layman’s terms… I mean*,* I’m reasonably aware of terminology … I had heard of cardiomyopathy*,* I was not exactly sure of how it works and how it affects me and things like that”* (P 8, age: 55–59).

#### Theme 4 – The value of home-visits

##### Supporting daily life activities

Most home visits were conducted by allied health professionals, who *“checked that everything … was safe”* (P 4, age: 75–79). These assessments were aimed at ensuring the safety of the person with HF, and the provision of practical support.

“*The first one [Occupational therapist] came when I was having the dizzy spells*,* and they got me the wheelie walker…They [Occupational therapists] found that sliding door was too high for me … I couldn’t get from the front veranda down into the carport to get in the car. She [Occupational therapist] measured up and made me a step to the carport and the ramp for the back of the house”.* (P 4, age: 75–79)

Participants spoke about the limitations of home-visits due to the perception of the individual’s role in symptom self-management, and that urgent care was still required to deal with exacerbations.

“*There’s not a lot of things anybody can do at home if someone is feeling slightly symptomatic*,* you either take your medication and it passes or it doesn’t pass or it gets worse when you call an ambulance*,* but there’s nothing you can do at home*” (P 15, age: 60–64).

##### Medical management in the home

Although participants had previous experience with allied health home-visits, many spoke about a preference for a physician or nurse to deliver care in the home setting but wondered if this was even feasible.

*“A lot of them are just medical issues … unless a doctor visited him at home*,* I don’t think there’s much else that could be done because it was like*,* you know*,* a urinary urgency and urinary tract infections and stuff… I can’t think of anything else apart from medical support that he would need or a nurse. I mean … that’s fantasy land stuff; I know that can’t happen but yeah.*” (C 2, age: 40–44).

Other participants who did not prefer home-visits expressed that their current situation did not warrant for support at home, as they felt capable of managing medication and had not experienced symptom exacerbations.

*“I just go to my appointments and if they [health professionals] change the medication*,* I just do that. And I haven’t really had any relapses or any problems with anything at home like doing that you know at home so I guess from my current situation*,* I don’t think I feel I would need any at home support at the moment”* (P 2, age: 50–54).

#### Theme 5 - Telehealth enhances care

##### Telehealth provides support and reassurance

Participants noted that telehealth, including the availability of telephone support from a dedicated health professional was an important component of home-based supportive care delivery. This is due to the value of telehealth in providing reassurance in their care-seeking decisions and symptom management. Participants valued telephoning a health professional and noted that it made them feel “*quite safe”* (P 8, age 55–59). This sentiment was also noted by carers who valued the psychological benefit of having access to telephone support from health professionals.

*“It builds confidence*,* but it decreases anxiety and those two combined are just a magic combination*” (C 9, age 55–59).

Telephone support was also considered important during symptom exacerbations, which allowed participants to clarify their doubts to avoid inconveniencing emergency and ambulance services.

“*Some people get wary and I am one of those people of calling the ambulance*”. (C 05, age 40–44)

Participants also noted that telehealth services enabled health professionals the opportunity to conduct remote monitoring and remind participants of appointments.

*“But sometimes I don’t go to my GP*,* I haven’t been probably for five weeks now*,* but she’ll probably call me because I’m due for a blood test in couple of weeks”* (P 13, age:65–69).

##### Nurse practitioners are well placed to provide telehealth

Amongst many participants, nurse practitioners were considered as the health professional best placed to provide support via telephone. This is due to nurse practitioners being perceived as available, accessible, and possessing the knowledge and skills to clarify participants’ queries.

“*I think it was also [Nurse Practitioner] that also said*,* if there was ever anything that I needed or would like to know more about or inquire about more information in order to help [Name of patient] from them*,* for me to get in touch any time”* (C 5, age 40–44).

Although participants spoke about the value of telephone support, they noted the limitation of it only being available during office hours on weekdays and a preference to ring outside of this time if it were possible.

*“I think it is only business hours [being able to phone health professionals for support] … but five days a week so I guess possibly I would have rang on the weekend if I could have but I have to wait till the Monday morning to call”.* (P 4, age 50–54)

This led to participants having to call the ambulance services after hours to seek support.

“*I think it’s just the day. And then if there’s anything outside of that*,* it’s pretty much ambulance”.* (C 5, age 40–44)

#### Theme 6 - A circle of care: Health professionals and social services supporting the individual with heart failure and carer

##### Continuity of care delivery

Participants noted they had experienced a long withstanding relationship with their health professional and appreciated a personal connection.

*“I think*,* I’m lucky to have a very good doctor [GP] who I’ve been with for 19 years*,* so*,* he’s has also been in the past someone that I can go and have a chat to*,* and then he can lead me into the direction because he knows me very well*,* he’s able to lead me into the right direction of the people that I need”* (C 05, age: 40–44).

Additionally, participants explained their frequent and regular interactions with health professionals which allowed for participants to feel that the health professional had a knowledge of them as an individual.

*“Although once again*,* as I said earlier*,* the fact that they’re [Heart support service] there and they’re we’ve been seeing them regularly*,* is enormous. I mean*,* it’s just such a relief. Just a huge relief”* (C 8, age:60–64).

*“Consistency has been really good with the cardiologist because she’s been seeing him all that time”* (C 10, age: 75–79).

While some participants noted they had frequent interactions, some expressed their preference for a consultation with their cardiologist more often.

*“a little bit more maybe to see him [cardiologist] more often instead of once every six months*,* once every three months”* (P 14, age:65–69).

##### GPs and nurse practitioners as care coordinators in a multidisciplinary team

Amongst the participants, GPs and nurse practitioners were the health professionals most frequently noted as the preferred care coordinators, who liaised with other health professionals and specialties.

*“…Doctor D [new GP] has contacted just about everybody that mom has come into contact with relating to heart failure at Hospital X and other issues at regional hospital Y. And she [new GP] has nailed it {stressing each word}! This woman is wonderful!* (C 9, age: 55–59)

A health professional such as a GP or nurse practitioner acting as a care coordinator led to enhanced communication amongst the individual with HF, their carer and the multidisciplinary team, which reduced feelings of overwhelm and enabled a greater knowledge and understanding of the HF illness.

*“She cried when we left there [signed off from HF clinic run by nurse practitioner] because she wasn’t gonna see her [Nurse practitioner B] again. But I can understand why. You ask and you get an answer. If the answer isn’t clear Nurse practitioner B will make a phone call and get the answer. And you left there [Nurse practitioner B’s clinic] with so much knowledge.* (C 9, age: 55–59)

However, some participants preferred not to visit the GP instead of the heart support services, as it was cumbersome and less helpful when navigating multiple morbidities and different specialists.

*“…when you’re regularly going to see the cardiologist and the kidney and the diabetes people*,* you’re generally having blood tests quite regularly*,* those three specialist areas will state what they want the medications to be. No GP is going to countermand what a specialist says. They’re [GP’s] not going to do it. So the only reason you go to a GP is they check your blood pressure… and they rewrite out the prescription renewals. They don’t really do anything else.”* (P 15, age: 60–64).

##### Community supports

Participants highlighted a need for additional knowledge of available community supports, as it was valuable in enhancing social connections which also led to a greater availability of supports and respite for both individuals with HF and carers.

*“When he could play sport and golf*,* he had outside interests where there’s not been a lot of input into that component of this rehab*,* like what he can do*,* what activities are available in the community*,* what community groups he could potentially link into. He hasn’t had any of that input*,* which I think would be really important as part of his rehab. And still allowing him to see that he can still do things he can still interact with people and*,* you know*,* still have that component of interactions*,* not just me that he needs to have near him for support”* (C 12, age: 55–59).

*“We haven’t really tapped into that. I don’t have an opinion on community only because there probably are lots of things out there for him*,* but we just don’t know about them”* (C 2, age: 40–44).

When asked about the type of support preferred while living with HF at home, participants expressed a desire for someone to assist with household tasks.

*“Probably there is somebody just to help you do your housework”* (P 10, age 75–79).

#### Theme 7 - Planning for future care

##### Various levels of planning had occurred

Individuals with HF and carers held diverse perspectives towards planning for the future. The unpredictability of HF led to individuals planning for the future differently, with some participants planning with their loved ones for an impending death.

*“He started*,* I think*,* mentally preparing himself that he wasn’t gonna be around for much longer*,* which then scared the heck out of us … so you know*,* he was having talk of ‘OK*,* you know*,* this is the finances*,* and this is this’ and I was like*,* wait*,* hang on a minute”* (C 5, age: 40–44).

However, other participants had a short-term plan in place which involved regular check-ups with their GP based on their needs, or a plan which involved resolving other immediate comorbidities.

*“Initially just checking in with his GP every six months when he needed his scripts done”* (C 6, age: 55–59).

*“…we’ve gotta get him through this pre-admission*,* then the surgery and then fingers crossed hopefully we’ll have a nice Christmas and then they’ll book him in for this other thing*,* for the heart…”* (C 8, age:60–64).

Some participants delayed planning for the future and instead preferred to deal with concerns as they arose, “…*I’ll cross that bridge when it comes”* (P 15, age: 60–64) and intended to plan if their condition worsened or changed.

*“…I mean*,* I don’t think we’re anywhere near there [severely ill and symptomatic] yet. So*,* there’s been no discussion of any plan*,* just regular checks with the GP and we’ll come and see the specialist and if there’s any changes*,* they’ll deal with them as they occur. That’s sort of it thus far”* (C 8, age: 60–64).

Some participants spoke about a lack of information being a barrier to enable planning for the future.

*“No [Discussion about future plan by the health care team]*,* just because we don’t know*,* there’s no point in making plans… you don’t make plans if you haven’t got all the information in front of you*,* you really can’t make definite plans”* (C 10, age: 70–74).

##### Quality of life matters

The need to be informed and engage in discussions on advance care planning and health directives were noted as important in knowing and respecting the wishes of their loved ones.

*“Yes*,* the yeah power of attorney…it is good to have someone because*,* especially when things happen to a loved one*,* different family members*,* different emotions…but the good thing is*,* I know what his wishes are and vice versa”* (C 5 age: 40–44).

Regardless of the extent of planning that had occurred, participants placed great value on the importance of quality of life over quantity of life.

*“That’s number one [quality of life]! On the top of the list {future care}. I don’t wanna be a burden and I think it’s important that you don’t want to be a burden on society”* (P 14, age: 65–69).

*“Quality of care over quantity of care means an awful lot… And the quality of life is not going to be worth a cracker*,* then there’s no point in mum existing. Because that’s not what she wants”* (C 3, age: 60–64).

## Discussion

This study describes the experiences, perspectives and preferences on palliative and supportive care at home for individuals with HF and their carers. The findings have important implications in optimising the design and delivery of home-based palliative and supportive care for this population. Understanding the experiences of individuals with HF and their carers provides insight into the types of support they may need and prefer [21, 22], in order to improve quality of life throughout the illness and also to guide policymakers in the development of patient and carer-centred interventions [7, 21].

Heart failure leads to many lifestyle changes for the individual, their carers and families. The possibility of HF symptoms worsening leads to anxiety and fear amongst individuals with HF and their carers [23, 24]. While individuals navigated emotional uncertainty in various ways, ongoing psychosocial support for the individual with HF, their carers and families are integral for improved quality of care [24].

Carers are vital to ensuring the wellbeing of individuals living with HF. However, the caregiving demands for individuals with HF are described as cumbersome by their carers, with carers experiencing physical, emotional and financial consequences, affecting their own quality of life, as well as those with HF [25]. Timely support to navigate these challenges is needed, including involving carers in care of the individual with HF in a team approach [26]. Delivering training to carers on HF caregiving tasks and conducting comprehensive assessments of carers’ wellbeing and their needs for social and financial support, could better support carers of individuals with HF [27, 28].

Telehealth for symptom management and disease monitoring is used in the fields of palliative care [29] and HF [15, 30] bringing health care to the patient’s home. In our study, the availability of telehealth provided reassurance for both individuals with HF and carers for symptom management, monitoring of the disease and in certain instances, to avoid an unwarranted trip to the emergency department. There is evidence that telehealth improves self-care behaviours, overall health-related quality of life and reductions in mortality and hospitalisations in the individuals living with HF [31]. Contact with a dedicated health professional, specifically a nurse practitioner, was preferred by most participants. Nurse-led telehealth interventions are a promising avenue to improve patient outcomes, and optimise resource utilisation [32] and hold promise in the delivery of HF palliative and supportive care [29]. Further research is needed on how to improve access to and delivery of out-of-hours telehealth services.

Home visits provide additional insight into the individual’s way of life [33]. Allied health services, including, but not limited to, occupational therapists and physiotherapists were among the most common health professionals to visit the homes of participants in our study. Home visits conducted by clinicians can enable a better understanding of the health and non-health factors of the patient [34]. Telehealth could be a solution to increasing access to medical support in the home, by linking medical professionals with individuals with HF and their carers, as well as nurses and allied health professionals to provide guidance around symptom management and remote medical monitoring [35]. A holistic approach to comprehensive care - coordinated across settings and disciplines – is central to supporting quality of life at home for individuals with HF [36]. A study exploring the psychosocial experiences of individuals with end-stage HF and their carers managing the disease at home recommended integrating community support services into home care, noting that providing information on these services can benefit both individuals with HF and their carers [23].

Health professionals play a crucial role in the delivery of high-quality palliative and supportive care. A trusting relationship between health professionals and those with HF and their carers supports the individual’s wellbeing and access to health care services [7, 37]. Individuals with HF and carers valued care coordinated by a health professional who understood their disease and knew them well [26, 38]. The importance of good communication is key for palliative and supportive care in HF in the home setting [10, 39, 40]. A systematic review exploring the unmet needs of patients and carers on home-based palliative care reported disjointed communication between health professionals as a contributor to unmet needs [40]. Disjointed communication hinders seamless delivery of care, causing confusion and a lack of confidence among individuals living with HF and carers. Thus, good communication involves transparency and the opportunity for clarification, which is crucial to understanding the disease trajectory of HF and providing emotional support and reassurance to those with heart failure and their carers. Furthermore, participants’ preferences for conversations on planning for future care could guide health professionals to plan and initiate a conversation as early on, at the time of diagnosis instead [41]. This could lead to a more empowered individual willing to be more engaged in their care management [21].

### Strengths and limitations

The qualitative methodology used in this study enabled an in-depth exploration of the research question from the perspectives of individuals with HF and their carers. The semi-structured open-ended interview guide, which was iteratively refined, allowed for exploration of the topic, which may not have been feasible if a survey had been used [18],

However, all carers that participated in this study were female, which may have influenced the perspectives represented in the findings. As participation was voluntary, this introduces a potential selection bias, whereby individuals who were more engaged or had the capacity to participate may have been overrepresented. Additionally, participants were recruited from two tertiary care hospitals within a single Australian region, which limits the transferability of the findings to other settings or populations. The findings should be considered within the context of the Australian healthcare system including its publicly funded model of healthcare. While generalisability is not the main aim of qualitative research, this study provides in-depth insights into experiences of individuals and carers that may be transferable to similar contexts.

## Conclusion

Our study provides guidance for designing and delivering home-based palliative and supportive care that aligns with the preferences and priorities of individuals living with HF and their carers. Understanding preferences for telehealth, home-visits, health information and improved communication, offers insights into how to provide individual and carer-centred care in accordance with their needs, leading to improved outcomes. Future research implementing interventions based on these findings could determine whether measurable improvements in outcomes are achievable.

## Supplementary Information

Supplementary Material 1.

## Data Availability

The dataset generated and/or analysed during the current study are not publicly available due to ethical considerations but is available from the corresponding author on reasonable request.

## Abbreviations

HF: Heart failure
GP: General Practitioner
