# Supplementary material for: Experiences, perspectives and preferences for home-based palliative and supportive care: a qualitative study of individuals with heart failure and their carers
Source: BMC Palliat Care. 2026 Apr 27;25:170. doi: 10.1186/s12904-026-02097-x (PMC13255324; doi:10.1186/s12904-026-02097-x)
Supplement: Supplementary file 1 — Supplementary Material 1. [file 12904_2026_2097_MOESM1_ESM.docx]

# Interview Guide for semi-structured interviews

Persons with heart failure and carers

- Thank you very much for agreeing to do this interview with me.
- My name is __________and I am a researcher with _______.
- Today, I’m interested in talking to you about care at home for persons with heart failure and what you think is important when receiving support at home.
- Some important things to note are
  - Our interview today will last about 45-60 minutes.
  - The conversation will be recorded as it will help me with my analysis for the research
  - You don’t have to answer any question that you don’t feel comfortable answering.
  - All information will be de-identified for research purposes, so your name or your address and other personal details will **NOT** be used in any published work.
  - I might take some notes while you speak to help me remember to ask more about something you mentioned.
  - If you feel any difficulty in breathing or any other discomfort during the interview, please let me know so we can stop and make sure you’re ok.
- Does this sound ok to you? Do you have any questions for me?
- Before we start the interview, I’d like to ask a couple of questions (Demographic form)
- I am going to **BEGIN RECORDING NOW** is that ok?

| **Question/s** | **Note taking space** |
| --- | --- |
| 1. **So to start off with, I am just going to ask a few general questions about your experience living with heart failure so could you tell me a bit about when and how you/the person you’re caring for was diagnosed with your/the heart condition?** |  |
| 1. **What has been your day to day life living with and managing your HF/ caring for a loved one living with HF?** |  |

| **Question/s** | **Note taking space** |
| --- | --- |
| 1. **What has your experience been of the health care you have received for your condition/your loved one’s condition?**   *Where have you/your loved one received care? (e.g. hospital, general practice, community settings)*  *How long have you/ your loved one been receiving that care?*  *Now, something that I am really interested in is I know that people with heart failure receive care in the hospital or when they see their cardiologist, but importantly, people spend most of their time at home and they have to manage their heart condition at home and maybe do things at home differently. And so I wanted to ask*  *Have you/your loved one had anyone (e.g. health care provider/ social worker etc.) visit you/your loved one at home?*   - *If yes, how was that experience?* - *If no, is this something you would like? What would that look like for you?* |  |
| So on that note, I am very interested in people living well with their heart condition at home because that’s where most people spend time, it’s not in the clinic or in the hospital, it’s at home.  This means making sure people with heart failure receive supportive care at home Supportive care for heart failure is something you can receive at any time and it’s about making sure you feel physically well, psychologically well, spiritually well and are socially connected. And this can be achieved in many ways, such as making sure your symptoms are managed, you know what medicines to take, you have a plan for the future and you have information. All the things to make sure you have a good quality of life living with heart failure and being at home.  Today I would like to talk about receiving this care at home   1. **From what I have described, what are your general thoughts about this?**   *Have you/your loved one had any experience receiving any supportive care at home? If yes, what was that like?*  *If no, is this something you would like? What would this look like for you?*   1. We know managing your/ your loved one’s symptoms at home by yourself and your loved one is a component of supportive care.   *What do you think about this?*  *What could be done so that your/ your loved one’s symptoms could be better managed at home in some way?*   1. Providing supportive care at home also involves specialities working together like cardiology and primary care like your/your loved one’s GP, nurses and physio.   *What do you think about this?*  *What is your experience with cardiologists and GPs involvement in your/your loved one’s care?*   1. When providing supportive care at home, communication with your/your loved one’s GP and your/your loved one’s cardiologist and team is needed.   *What has been your experience of communication between the team members?*  *What are your thoughts on how to support good communication?*     1. Planning for your/ your loved ones care is a part of supportive care.   *Looking back on the care you received what do you think would have been more helpful to plan your care?*  *What would have been helpful to enable you/ your loved one to be involved in the decisions on the care received and to feel more involved in your/ your loved one’s care?*  *What decisions about your/your loved one’s care would you like to be involved in?*   1. Part of supportive care is also education about heart failure for you and your loved one.   *What information and education would be or would have been helpful to you? When would this have been helpful and how could this education be provided? What could be done to help provide education in the home setting?*  *What other resources can be useful when obtaining care at home for you/ your loved one? What would this look like for you?*  So we have spoken about managing symptoms at home, the cardiology and GP working together, education on HF and discussions about future care at home and resources.  *What are the most important aspects to you? What makes these things most important? What are your thoughts on how and when these aspects of care could be provided in the home?*  *If you were to feel more unwell than you are usually what support would you like to have? From whom would you like ?*  *If you were to feel well than usual, what support would you like to have?*    Is there anything you’d like to add.  Thank you very much for your time and talking to me...  -End of Guide- |  |
